# Supplementary material for: Functional Inactivation of PAX4 Results in Disrupted Endocrine Pancreas Development and Neonatal Diabetes in Pigs
Source: Int J Mol Sci. 2026 Jun 23;27(13):5651. doi: 10.3390/ijms27135651 (PMC13361469; doi:10.3390/ijms27135651)
Supplement: Supplementary file 1 [file ijms-27-05651-s001.zip › ijms-4362878-supplementary.pdf]

Supplementary Information

Functional inactivation of *PAX4* results in disrupted endocrine pancreas development and neonatal diabetes in pigs

Ravikanthreddy Poonooru[1], Ki-Eun Park[1], Amanda Schmelzle[1], and Bhanu P. Telugu[1*]

[1] Division of Animal Sciences, University of Missouri, Columbia, MO 65211.

*Correspondence: telugub@missouri.edu; 920 E. Campus Dr., 105 ASRC, Columbia, MO 65211

This PDF file includes:

Supplementary Figures (S1 to S9)

Figure S1. CRISPR/Cas9 gRNA design, embryo development outcomes, and sequence validation for targeting porcine *PAX4*.

Figure S2. Predicted 3D structures of wild-type and mutant porcine *PAX4* proteins.

Figure S3. Sanger sequencing of *PAX4* alleles in WT and BE4-edited piglets.

Figure S4. SnRNA-seq reveals endocrine-cell depletion and broad transcriptional disruption in *PAX4*-KO neonatal pancreas.

Figure S5. Bulk RNA-seq shows loss of endocrine-cell markers and activation of diabetes-associated transcriptional signatures in *PAX4* KO neonatal pancreas.

Figure S6. Gene ontology enrichment analysis of genes upregulated in PAX4-KO neonatal pancreas

Figure S7. Representative H&E histology and insulin immunohistochemistry of WT and *PAX4*-KO neonatal pancreas

Figure S8. Immunofluorescence staining of WT neonatal pancreas

Figure S9. Immunofluorescence of *PAX4*-KO pancreas

Supplementary Tables (S1-S3)

Table S1. Overview of Guide RNA Sequences and Screening Primers

Table S2. Antibody Panel and Working Dilutions

Table S3. Statistical analysis plan and outputs for phenotypic, survival, and serum biochemistry data

A

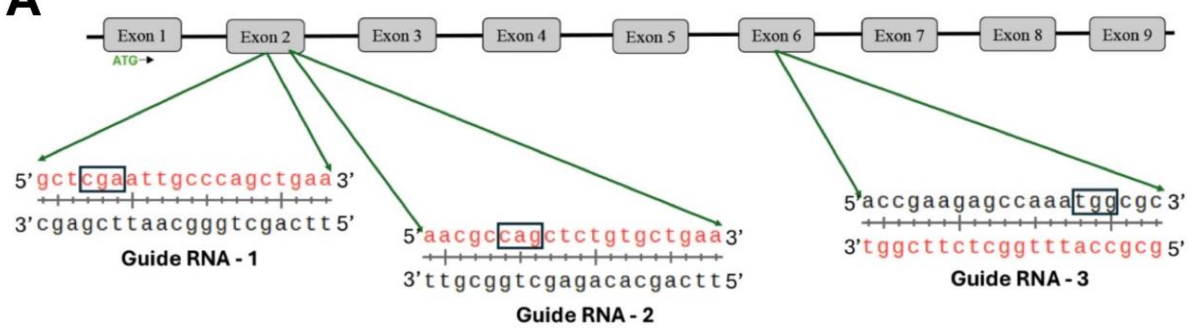

B

Guide RNA tested	No. of oocytes	No. of blastocysts	Blastocyst percent	KO percent
Wild type	30	19	63.33	-
Gude #1	30	20	66.67	0 %
Gude #2	30	18	60.00	0 %
Gude #3	30	20	66.67	80 %

C

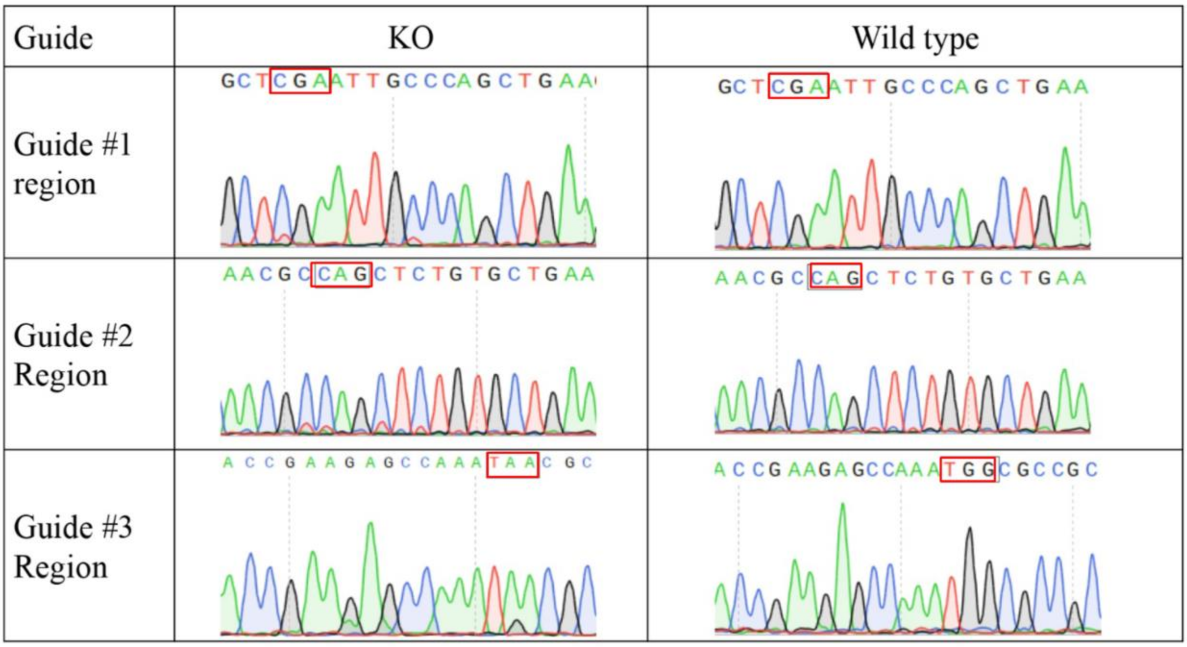

Figure S1. Base editor sgRNA design, embryo development outcomes, and sequence validation for targeting porcine *PAX4*. (A) Targeting map of three CRISPR gRNAs across the porcine *PAX4* locus (B) CRISPR guide selection: Blastocyst yield and knockout efficiency of porcine zygotes (C) Sequence verification of CRISPR edits at three gRNA sites in the porcine *PAX4* locus.

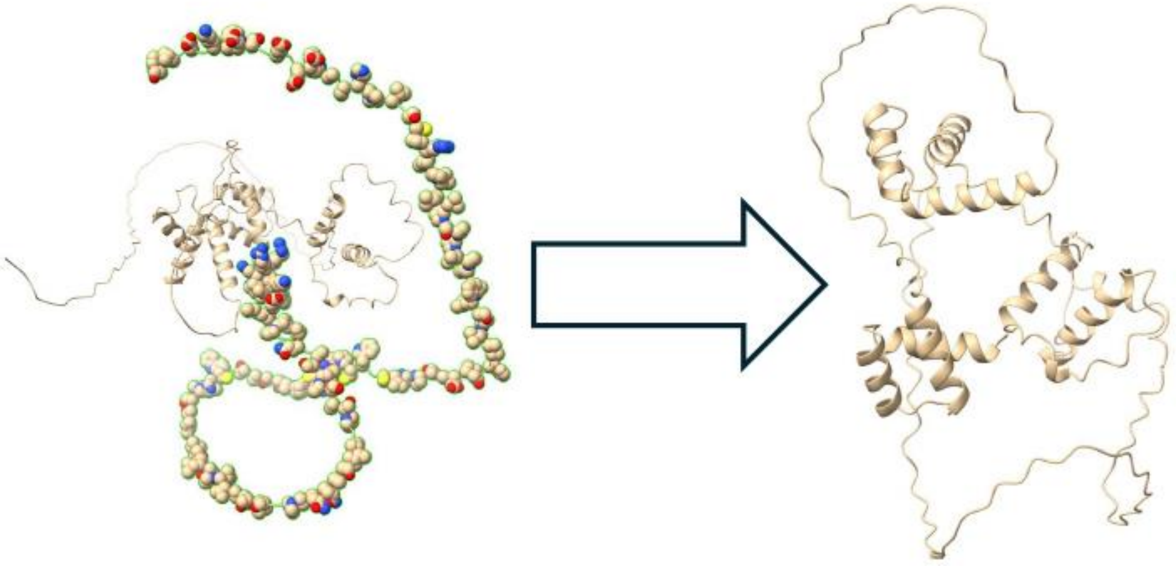

Figure S2. Predicted 3D structures of wild-type and mutant porcine PAX4 proteins. The structure of *PAX4* wild-type (**Left**) shows a well-folded region comprising several α-helices, including a portion of the homeobox domain responsible for DNA binding. This domain is highlighted using space-filling (spherical) representation to emphasize the region that is expected to be structurally and functionally critical. In contrast, the *PAX4* mutant (**Right**), generated by introducing a premature stop codon, shows a loss of the homeobox domain and associated structural elements. The absence of this region is expected to result in the loss of the DNA-binding ability of the protein and compromise its role in transcriptional regulation.

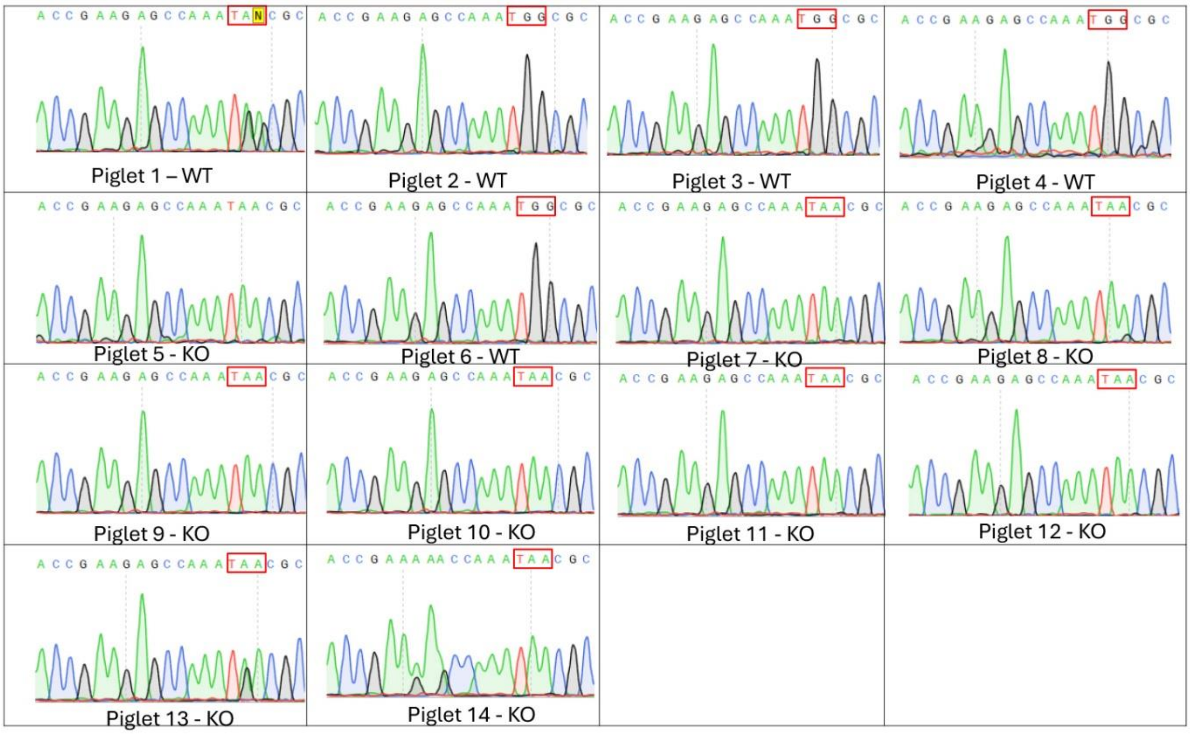

Figure S3. Sanger sequencing of *PAX4* alleles in WT and BE4 edited piglets**.**

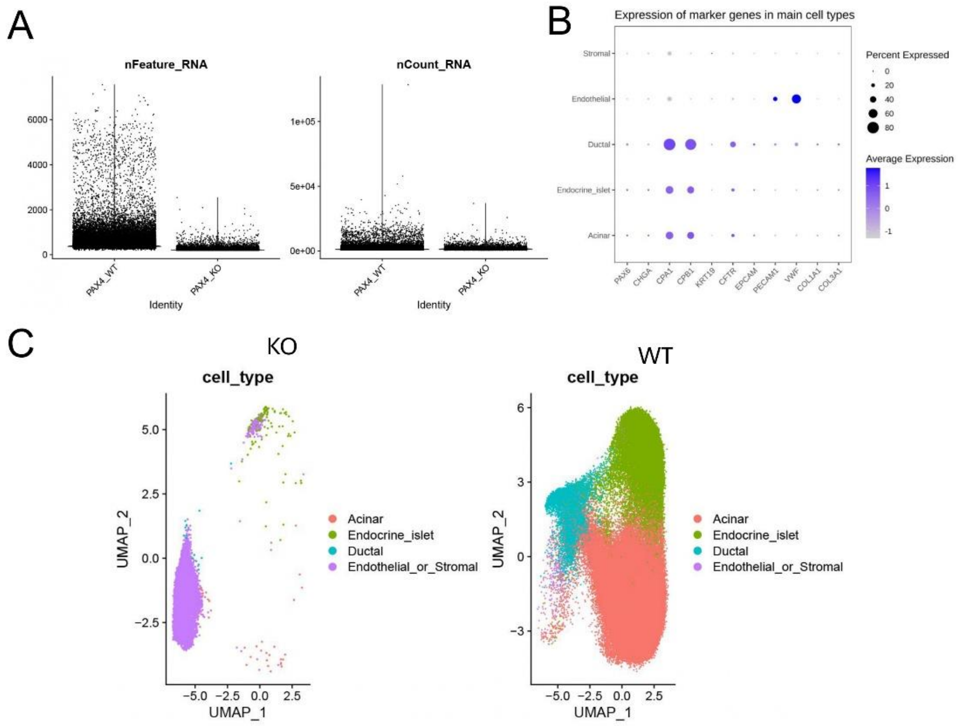

Figure S4. SnRNA-seq reveals endocrine-cell depletion and broad transcriptional disruption in *PAX4*-KO neonatal pancreas.
(A) QC metrics. (B) Cell-type marker validation. (C) UMAP by genotype and cell type

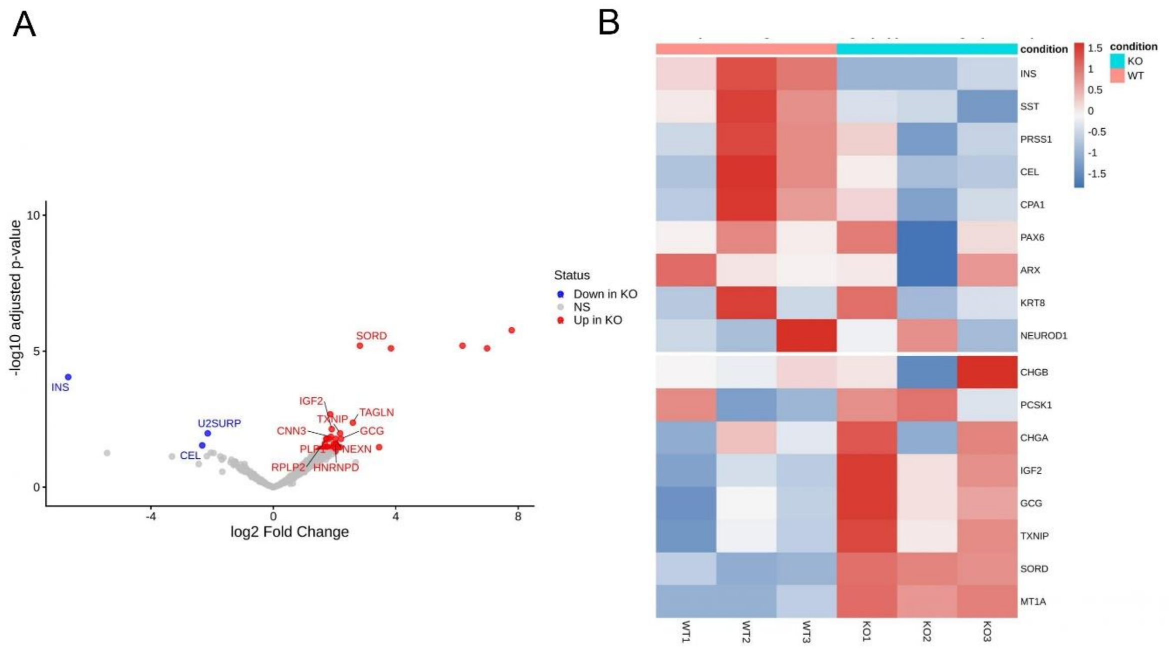

Figure S5. Bulk RNA-seq shows loss of endocrine-cell markers and activation of diabetes-associated transcriptional signatures in *PAX4*-KO neonatal pancreas. (A) Volcano plot. (B) Targeted heatmap.

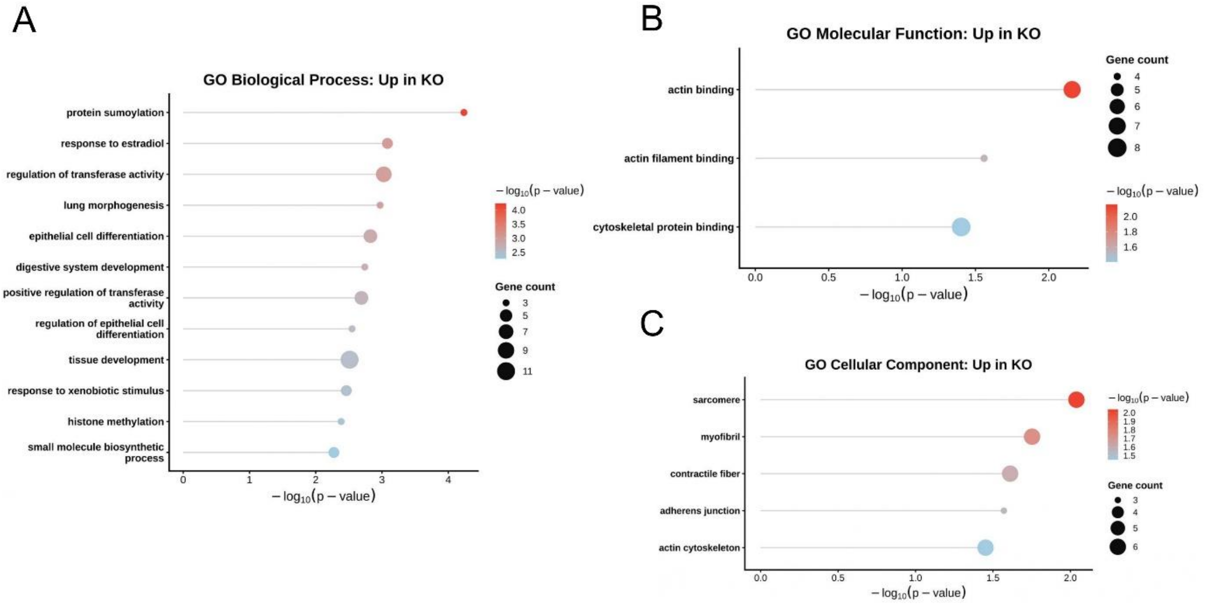

Figure S6. Gene ontology enrichment analysis of genes upregulated in *PAX4*-KO neonatal pancreas.
(A) GO Biological Process enrichment. (B) GO Molecular Function enrichment. (C) GO Cellular Component enrichment

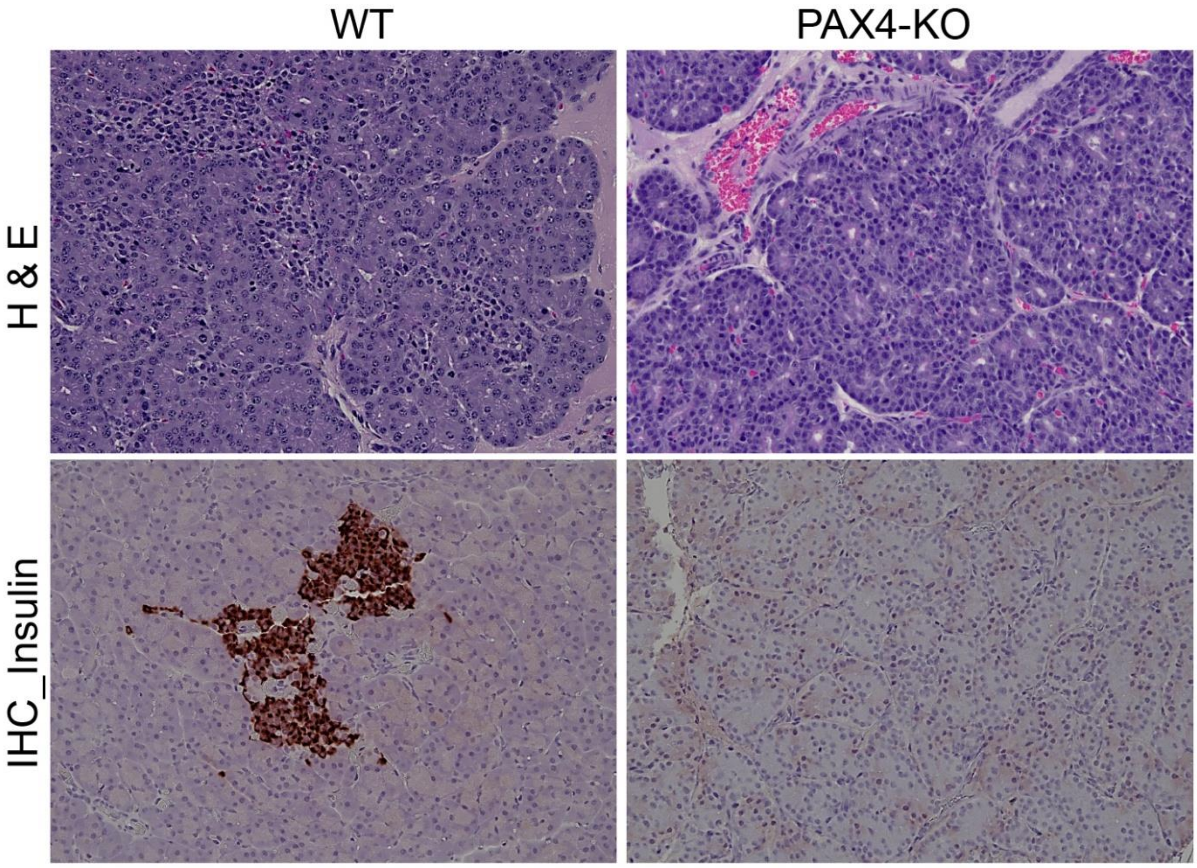

Figure S7. Representative H&E histology and insulin immunohistochemistry of WT and *PAX4*-KO neonatal pancreas showing loss of insulin-positive islets in *PAX4*-KO

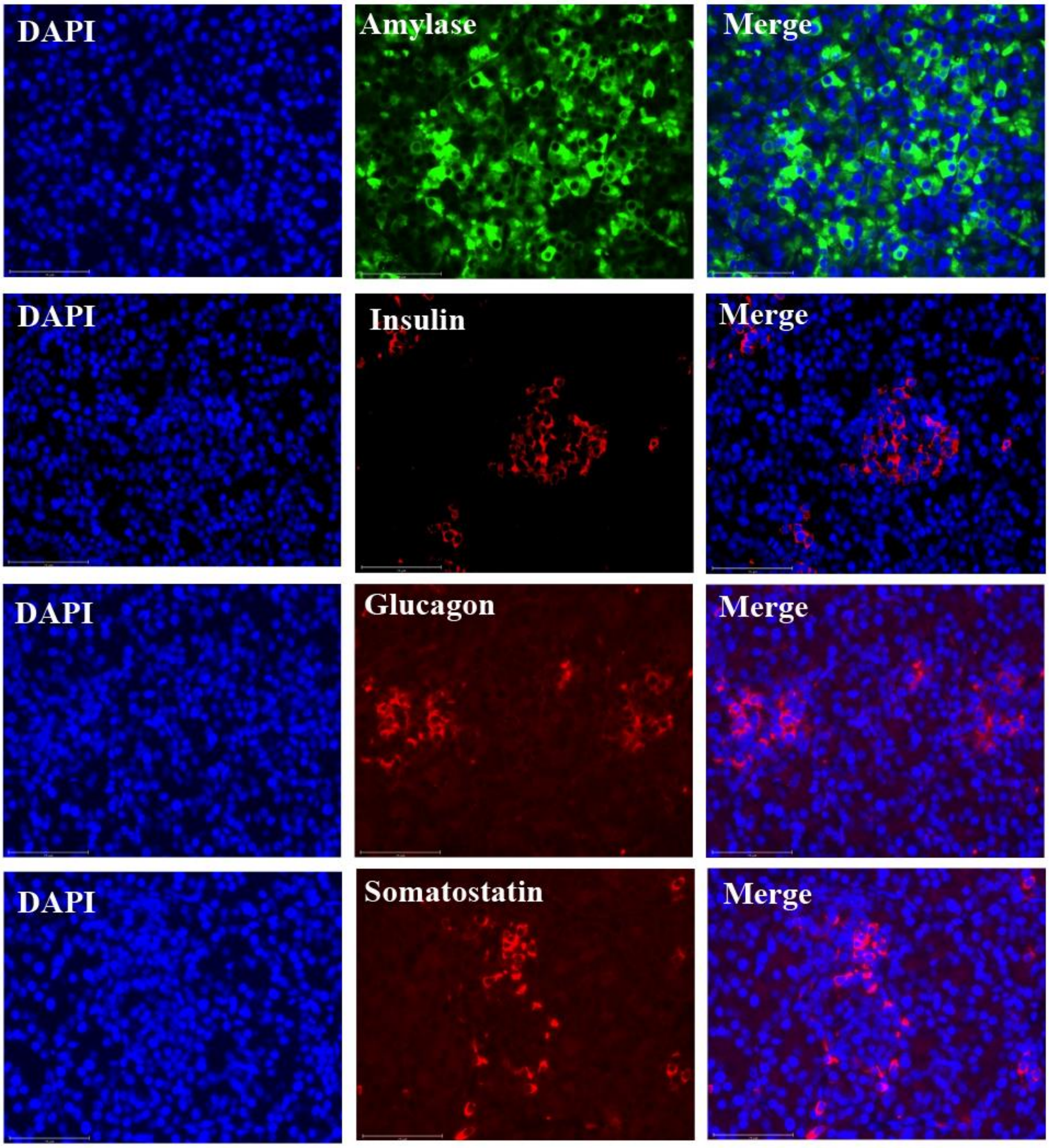

Figure S8. Immunofluorescence staining of WT neonatal pancreas showing exocrine amylase- and endocrine hormone-positive cells (insulin, glucagon, somatostatin; 40X)

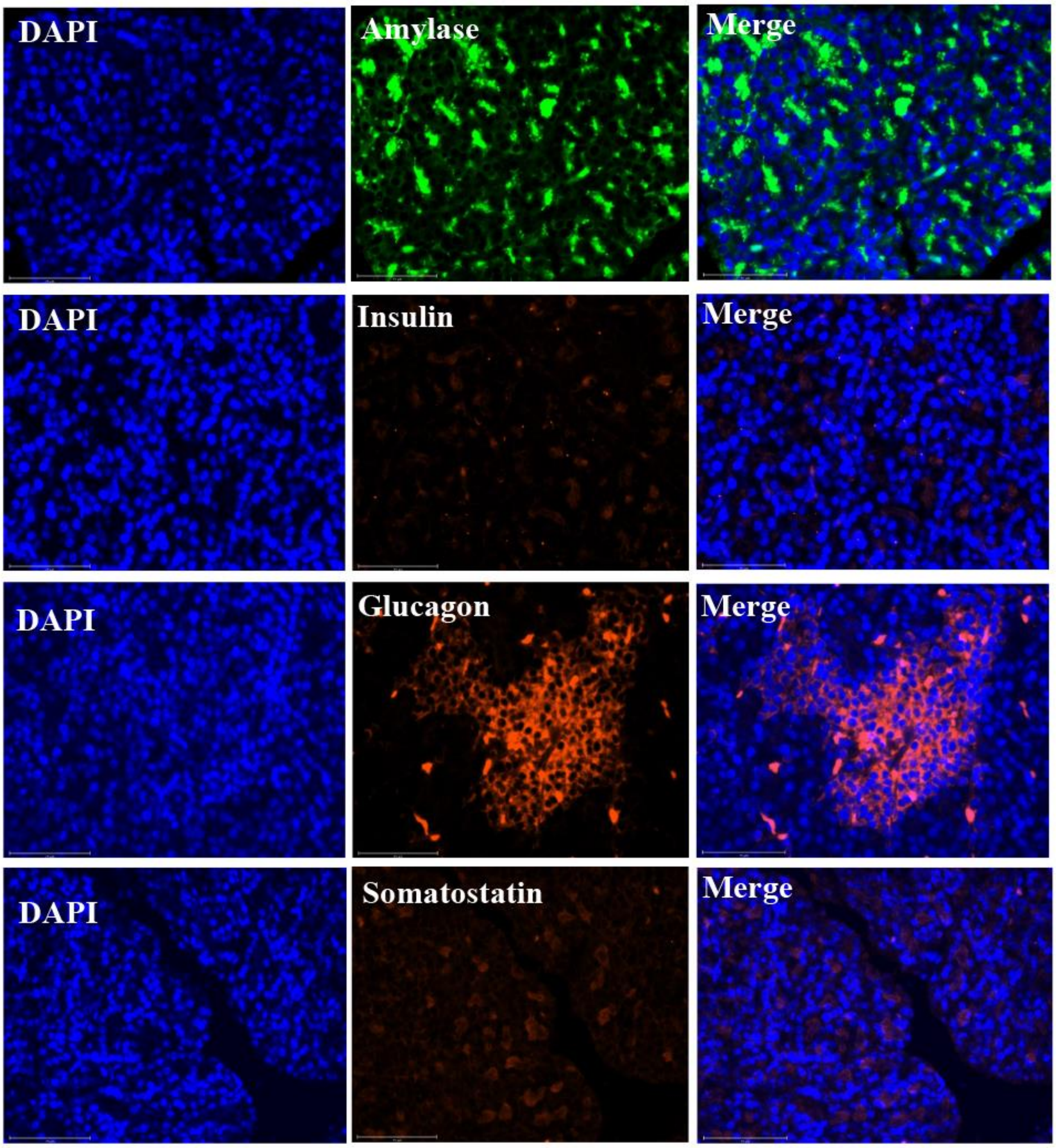

Figure S9. Immunofluorescence of *PAX4*-KO pancreas showing preserved amylase staining with loss of insulin- and somatostatin-positive endocrine cells (40X).

Note: The secondary antibodies used for glucagon and somatostatin in Supplementary figures 8 and 9 are different from those used in the main text: glucagon and somatostatin were detected with donkey anti-rabbit (Life Technologies, A10042).

Table S1. Overview of Guide RNA Sequences and Screening Primers

	Guide RNA/Primer	Sequence (5′-3′)
	Guide RNA #1	GCTCGAATTGCCCAGCTGAA
	Guide RNA #2	AACGCCAGCTCTGTGCTGAA
Guide RNA #1 and #2	Outer_Forward	CCTTACCTGTTCCTGCTTCCTA
	Outer_Reverse	TGCCAAGTACAAAGGGATCTTC
	Inner_Forward	GCAAGATCCTAGGGCGTTACTA
	Inner _Reverse	GTGGCACTTACACTGGGAGTCT
	Guide RNA #3	GCGCCATTTGGCTCTTCGGT
Guide RNA #3	Outer_Forward	AGCAGAAATGAGACAAGAGCCT
	Outer_Reverse	TGTGTAGCCCAGCATTTAACAC
	Inner_Forward	CAGGTCCGGTGAGAACAGTAG
	Inner _Reverse	AGAGGATAGGTGAGTGGAGTGG

Table S2. Antibody Panel and Working Dilutions

Primary Antibody				
S. No.	Antibody	Host	Dilution tested	Catalogue detalis
1	Anti_insulin	Guinea pig	1:150	DAKO (A0564)
2	Anti_glucagon	Rabbit	1:200	Sigma Aldrich (ZRB1115)
3	Anti_Somatostatin	Rabbit	1:300	DAKO (A0566)
4	Anti_Amylase	Goat	1:150	Santa Cruz Biotech (SC-12821)
Secondary Antibody				
S. No.	Antibody	Species	Catalogue details	
1	Anti_insulin	Donkey anti_guinea pig	Jackson immunoResearch (706-165-148)	
2	Anti_glucagon	Goat anti_rabbit	Abcam ab6721	
3	Anti_somatostatin	Donkey anti_rabbit	Novus Biologicals (NBP 1-75638)	
4	Anti_Amylase	Donkey anti_goat	Novus Biologicals (NBP 1-74824)	

Table S3. Statistical analysis plan and outputs for phenotypic, survival, and serum biochemistry data

S. No.	Parameter	Normal Distribution (Shapiro wilk's test)	Homoscedasticity (Lavene's test)	Statistical analysis	Statistical output
1	Body weight	Yes (P>0.05)	Yes (P>0.05)	ANCOVA	B/w groups<br>Genotype - P<0.001<br>Timepoint - P=0.176<br>Interaction - P<0.05<br>Within the group<br>WT (Timepoints) - P<0.01<br>KO (Timepoints) - P<0.01
2	Body Condition Score (BCS)	No (P<0.05)	No (P<0.05)	Aligned Rank Transform method	Genotype - P<0.001<br>Timepoint - P<0.001<br>Interaction - P=0.08
3	Blood Sugar (On-farm testing)	Yes (P>0.05)	Yes (P>0.05)	ANCOVA	Genotype - P<0.001<br>Timepoint - P<0.001<br>Interaction - P<0.05
4	Survival curve	-	-	Mantel–Cox test	Genotype - P = 0.033 (<0.05)
5	Serum components	Unequal and small groups		Kruskal-Wallis test	-
